# Time-Resolved Profiling of Aroma-Active Compounds During the Persistence of Empty Cup Aroma in Soy Sauce Aroma Type Baijiu

**DOI:** 10.3390/foods15162906

**Published:** 2026-08-19

**Authors:** Dan Qin, Xiangrong Fan, Jiawen Duan, Lin Zhu, Jiaqi Liang, Jingcheng Liu, Yanyan Zhang, Xiaoxue Chen, Hehe Li

**Affiliations:** 1 Food Laboratory of Zhongyuan, China Agricultural University, Beijing 100193, China; flyingqwfy@126.com (D.Q.); aggiefxr@163.com (X.F.); chen.xx@cau.edu.cn (X.C.); 2 Key Laboratory of Geriatric Nutrition and Health, Ministry of Education, Beijing Technology and Business University, Beijing 100048, China; duanjiawen11@126.com; 3 Department of Flavor Chemistry (150h), Institute of Food Science and Biotechnology, University of Hohenheim, Fruwirthstraße 12, 70599 Stuttgart, Germany; zhulin601@nwafu.edu.cn (L.Z.); jiaqi.liang@uni-hohenheim.de (J.L.); yanyan.zhang@uni-hohenheim.de (Y.Z.); 4 School of China Alcoholic Drinks, Luzhou Vocational and Technical College, Luzhou 646000, China; liujingcheng1980@126.com; 5 University-Enterprise Joint Innovation Base for Applied Brewing Engineering Technology of Sichuan Provincial Education Department, Luzhou 646000, China

**Keywords:** soy sauce aroma type baijiu, empty cup aroma, aroma extract dilution analysis, stir bar sorptive extraction, aroma-active compounds, boiling point

## Abstract

Empty cup aroma is a distinctive quality attribute of soy sauce aroma type baijiu (SSB), but the compounds responsible for its long persistence and their changes over time are still not well understood. Previous studies have mainly focused on key empty cup odourants at single time points. In this study, a typical commercial SSB was selected and monitored for 30 days using aroma extract dilution analysis (AEDA), time-resolved odour monitoring, residue peak area analysis and correlation with boiling point. AEDA revealed 65 aroma-active regions, including 63 in SSB, 36 in the empty cup and 34 shared by both. After emptying, the flavour dilution profile changed markedly: fruity esters and volatile aldehydes became less prominent, whereas sotolon showed the highest FD factor in the empty cup (FD 2048), and several acids and pyrazines also became prominent. The number of detectable regions increased from 43 at 0 h to 49 at 6 h, and then gradually decreased to 14 at day 30. Persistence was strongly correlated with boiling point (Spearman’s ρ = 0.7465, *p* < 0.0001, *n* = 32). Peak area profiles also showed two general patterns. Low-boiling esters, aldehydes and dimethyl trisulfide decreased rapidly, whereas organic acids, pyrazines, phenylethyl alcohol, γ-nonanolactone and ethyl hexadecanoate showed delayed maxima or non-monotonic changes. These results suggest that persistent empty cup aroma is not simply a residual form of the original baijiu aroma but develops through selective retention and temporal shifts in the contribution of different odourants.

## 1. Introduction

Baijiu is a traditional Chinese distilled spirit and one of the six major distilled spirits worldwide [1]. It is commonly classified into 12 aroma types: soy sauce, strong, light, rice, sesame, chixiang, texiang, complex, herb-like, fengxiang, laobaigan and fuyu [2,3,4]. Among these, soy sauce aroma type baijiu (SSB) is particularly complex in composition, partly because of its distinctive “12,987” production process [5]. This process involves one annual production cycle, two feedings, nine steamings, eight fermentation rounds and seven distillation rounds. The resulting SSB is characterised by a pronounced yet elegant sauce-like aroma, a mellow palate and a long aftertaste [6,7]. One of its distinctive sensory attributes is the persistent empty cup aroma, referring to the aroma that remains perceptible after the baijiu has been poured out and the cup is left standing for a period of time [8]. Persistent empty cup aroma is regarded as an important sensory quality attribute of SSB [6,7]. During tasting, empty cup aroma is considered alongside colour, taste and in-mouth aroma, but the trace odourants responsible for its persistence remain difficult to characterise.

Early studies of empty cup aroma were largely descriptive and focused on identifying residual volatile compounds using static headspace sampling coupled with gas chromatography-mass spectrometry (GC-MS) [9]. Esters, aldehydes and organic acids were detected, but their low concentrations in the empty cup made further characterisation difficult. Vacuum-assisted sorbent extraction later improved chemical coverage, with 81 compounds, including lactic acid, ethyl hexadecanoate and hexanoic acid, detected in the empty cup aroma of SSB [8]. Molecular sensory analysis further linked these residual compounds to their sensory relevance. Gas chromatography–olfactometry (GC-O) combined with aroma extract dilution analysis (AEDA) identified sotolon, phenylethyl alcohol, p-cresol and 2,3-dimethyl-5-ethylpyrazine as key contributors to empty cup aroma, with sotolon proposed as a characteristic marker [10,11]. In situ headspace sampling has also been used to monitor natural evaporation and to distinguish the geographical origin of baijiu based on empty cup profiles [12]. Other studies have explored the physicochemical factors underlying empty cup aroma. Lactic acid has received particular attention because of its high abundance in the empty cup [8], as well as its ability to interact with aroma compounds in Baijiu [13,14]. It can lower the olfactory detection threshold of sotolon and thereby enhance its sensory contribution [10]. Lactic acid and ethyl lactate can also affect the release of ethanol and other odourants through hydrogen bonding and competitive association in ethanol–water matrices [15,16]. These findings suggest that empty cup aroma involves selective retention driven by molecular interactions rather than the simple evaporation of the residual baijiu.

Despite these advances, it remains unclear whether the odourants that are most potent immediately after emptying are also the most persistent. Volatility is likely to influence persistence [16,17], but much of the available evidence comes from simplified model systems. Zhao et al. [17] monitored ester release from model baijiu using headspace solid-phase microextraction (HS-SPME) coupled with GC-MS and found that low-boiling esters declined progressively, whereas higher-boiling esters first increased and then decreased. However, authentic SSB contains many aroma-active compounds other than esters, including acids, alcohols, nitrogen heterocycles, and lactones. These compounds may interact differently with the matrix, and their temporal behaviour therefore cannot be predicted from ester-based model systems alone.

Analytical sensitivity is another challenge because empty cup residues contain fewer aroma-active compounds and at lower concentrations than the original baijiu. Stir bar sorptive extraction (SBSE) uses a stir bar coated with a substantially larger volume of polydimethylsiloxane (PDMS) than conventional solid-phase microextraction fibres, providing higher extraction efficiency and sensitivity for trace-level volatiles [18,19]. SBSE is also solvent-free and directly compatible with thermal desorption GC-O/GC-MS analysis. These features make SBSE well suited for detecting low-abundance volatile compounds [18,19,20]. SBSE has also been successfully applied to the analysis of volatile compounds in soy sauce aroma type baijiu [21]. We therefore used SBSE throughout this study.

Most studies of authentic SSB, however, have focused on individual time points. It therefore remains unclear whether the odourants that dominate immediately after emptying are also the most persistent, and how the overall aroma profile changes over time. To address these questions, we first compared the aroma-active profiles of SSB and the empty cup using GC-O/AEDA and then monitored individual odourants at multiple time points over 30 days using SBSE with odour detection and residue peak area measurements. We also examined the relationship between odourant persistence and boiling point as an indicator of volatility and characterised their temporal patterns. These analyses aimed to identify the main odourants associated with persistent empty cup aroma and to determine how their detectability and abundance change over time.

## 2. Materials and Methods

### 2.1. Samples

A commercial SSB sample (53% alcohol by volume, ABV) was obtained from a soy sauce aroma type Baijiu production enterprise in Renhuai, Guizhou Province, China, and used in this study. The replicated time-course experiment was designed to characterise temporal changes within this sample rather than variation among producers, batches, ageing periods or distillation regimes.

### 2.2. Chemicals

Analytical standards (≥95% purity) of 2-methylpropanal, ethyl acetate, acetal, 3-methylbutyraldehyde, ethyl 2-methylpropanoate, ethyl butanoate, ethyl 2-methylbutanoate, ethyl 3-methylbutanoate, 2-methyl-1-propanol, 3-methylbutyl acetate, ethyl pentanoate, ethyl 4-methylpentanoate, 3-methyl-1-butanol, ethyl hexanoate, 1-octen-3-one, 2,6-dimethylpyrazine, ethyl lactate, dimethyl trisulfide, 2-ethyl-6-methylpyrazine, trimethylpyrazine, 2-furanmethanethiol, acetic acid, 2,3-dimethyl-5-ethylpyrazine, tetramethylpyrazine, 2,3-butanediol, 2-methylpropionic acid, butanoic acid, 2-acetyl-5-methylfuran, 1-nonanol, 3-methylbutanoic acid, pentanoic acid, ethyl phenylacetate, *β*-damascenone, hexanoic acid, geranylacetone, ethyl 3-phenylpropanoate, phenylethyl alcohol, heptanoic acid, 2-acetylpyrrole, γ-nonanolactone, octanoic acid, p-cresol, nonanoic acid, sotolon, ethyl hexadecanoate, n-decanoic acid, benzeneacetic acid, and hydrocinnamic acid, together with n-alkanes (C7–C40) were purchased from Sigma-Aldrich Chemie (Taufkirchen, Germany), Alfa Aesar (Kandel, Germany), Carl Roth (Karlsruhe, Germany), J&K (Altdorf, Germany) and VWR International GmbH (Darmstadt, Germany).

### 2.3. Aroma Extraction by Stir Bar Sorptive Extraction (SBSE)

SSB (53% ethanol by volume) was diluted with water to 10% (*v*/*v*) ethanol. A 10 mL aliquot of the diluted sample and 3.5 g NaCl were transferred to a 20 mL headspace vial. Volatiles were extracted with a polydimethylsiloxane (PDMS)-coated Twister^®^ (10 mm length; 0.5 mm PDMS thickness, Gerstel, Mülheim an der Ruhr, Germany) at 750 rpm and room temperature for 90 min. The stir bar was then removed, rinsed with deionised water, dried with lint-free tissue and placed in a conditioned thermal desorption unit liner. The thermal desorption programme was held at 40 °C for 1 min, followed by heating to 150 °C at 120 °C/min and holding for 10 min. Desorption was performed in solvent vent mode with splitless transfer to the cooled injection system at 250 °C. Cryofocusing was performed at −100 °C for 1 min, followed by heating to 175 °C at 720 °C/min and holding for 5 min before transfer to the GC column [22].

For empty cup aroma extraction, 20 mL SSB was transferred to a 40 mL headspace vial and held for 2 min. The baijiu was then discarded, and the residual sample remaining in the vial was evaporated to dryness under a stream of nitrogen. A PDMS-coated Twister^®^ stir bar was subsequently placed directly in the vial containing the dried residue and rotated at 300 rpm at room temperature for 90 min to extract the residual odourants. The stir bar was then subjected to thermal desorption under the conditions described above.

Persistence experiments were conducted in a sensory evaluation room at 25 ± 2 °C. Forty-millilitre headspace vials were used to simulate empty cups. Each vial received 20 mL SSB, which was held for 2 min and then discarded. The time at which the baijiu was discarded was defined as 0 h. Samples were analysed at 0, 6, 12, 24, 36 and 48 h and at 3, 5, 10, 15, 20, 25 and 30 d. At each of the 13 time points, three replicate vials were randomly selected for SBSE followed by GC-O-MS analysis. An aroma-active region was considered detected when it was perceived in at least two of the three replicates. Assessors rated the intensity of each aroma attribute on a scale from 0 to 4, where 0 indicated imperceptible, 1 weak, 2 moderate, 3 clearly perceptible and 4 strong. Mean intensity scores from triplicate evaluations were used to construct the temporal profiles. Persistence was defined as the last time point at which an aroma-active region met the detection criterion. Values reported as ≥30 d indicate that the region remained detectable at the final sampling point and were therefore right-censored by the 30 d experimental window.

### 2.4. Identification of Aroma-Active Compounds by GC-O-MS

Odourants were analysed using an Agilent 7890B gas chromatograph equipped with a polar DB-WAX column (30 m × 0.25 mm internal diameter; 0.25 μm film; Agilent Technologies, Santa Clara, CA, USA). The GC was coupled to an Agilent 5977B mass selective detector and an ODP 3 olfactory detection port (ODP3, Gerstel, Mülheim an der Ruhr, Germany). Helium (grade 5.0; Westfalen, Münster, Germany) was used as the carrier gas at 1.62 mL/min. The column effluent was split 1:1 between the mass spectrometer and the olfactory port using a μFlowManager splitter (Gerstel, Mülheim an der Ruhr, Germany) at a column outlet pressure of 20 kPa. The oven temperature was held at 40 °C for 3 min, increased to 240 °C at 5 °C/min and held for 10 min. The septum purge flow was 3 mL/min, the inlet temperature was maintained at 250 °C, and samples were injected at a split ratio of 2:1. The GC-MS transfer line was maintained at 250 °C. Mass spectra were acquired in full-scan mode over *m*/*z* 35–350 with a 3 min solvent delay and electron ionisation at 70 eV. The ion source and quadrupole temperatures were maintained at 230 and 150 °C, respectively. The olfactory port transfer line and mixing chamber were maintained at 250 and 150 °C, respectively, with nitrogen (grade 5.0; Westfalen) used as the make-up gas. For GC-O identification, three experienced assessors evaluated each sample in duplicate. All assessors had prior experience in GC-O analysis of food and completed a calibration session using a subset of reference standards before the formal analyses to familiarise them with retention times and standardise aroma descriptors. Aroma-active compounds were identified by comparing their retention indices on the DB-WAX column, aroma descriptors and mass spectra with those of authentic standards when available. Compounds without authentic standards were tentatively identified based on mass spectra, retention indices and aroma characteristics. Retention indices were calculated using a C7–C40 n-alkane series.

### 2.5. Aroma Extract Dilution Analysis Combined with SBSE

AEDA was performed on SBSE extracts of SSB and empty cup samples by progressively increasing the split ratio during thermal desorption. After extraction, the odourant-loaded stir bars were thermally desorbed at successive split ratios of 1:2^n^ until no odour was detected. The FD factor of each odourant was defined as the highest split ratio at which it remained detectable. A split ratio of 1:2048 was the practical upper limit of the dilution series under the thermal desorption conditions used. Therefore, an FD factor of 2048 represents the highest dilution level tested rather than the actual detection limit of odourants that remained detectable at this level. At each dilution level, three replicate samples were analysed. An odourant was considered detectable when it was perceived in at least two of the three replicates, and its FD factor was assigned as the highest dilution meeting this criterion.

### 2.6. Statistical Analysis

FD comparison plots were generated using OriginPro 9.0 (OriginLab, Northampton, MA, USA), and temporal peak area profiles were generated using GraphPad Prism 10 (GraphPad Software, Boston, MA, USA). Boiling point values and their sources are provided together with the persistence results (Section 3.3). Values were rounded to the nearest degree Celsius and used for correlation analysis. Spearman’s rank correlation was used to assess the association between odourant persistence and boiling point. For the statistical analysis, observations reported as ≥30 d were assigned a value of 30 d, corresponding to the final sampling point. Statistical significance was set as *p* < 0.05.

## 3. Results and Discussion

### 3.1. Aroma-Active Compounds in SSB and Empty Cup Aroma

**Figure 1:**
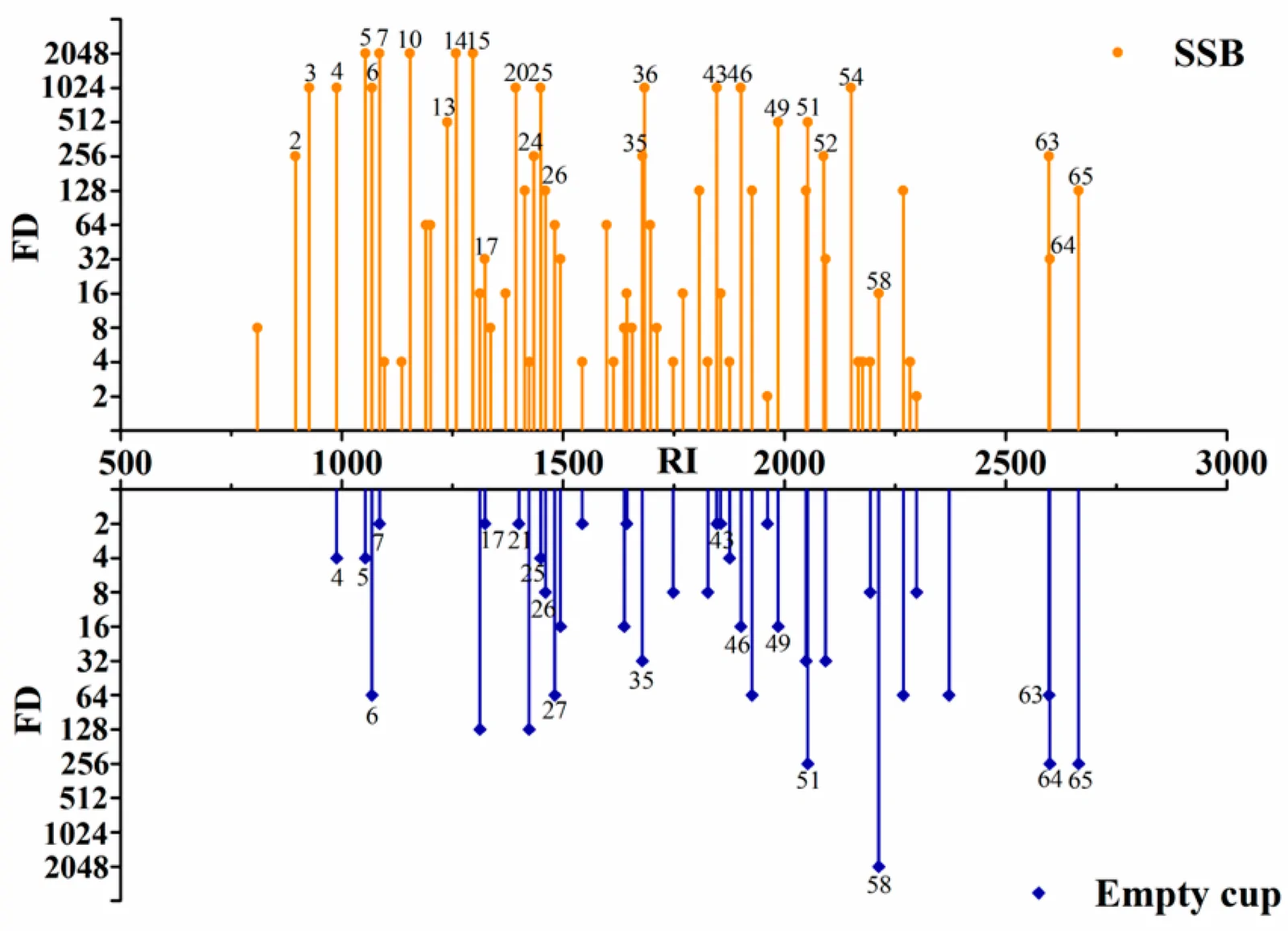
Comparative AEDA profiles of aroma-active regions in SSB and the empty cup. Numbers indicate the compound identifiers listed in Table 1.

AEDA detected 65 aroma-active regions across the two samples, including 63 in SSB, 36 in the empty cup and 34 shared by both (Table 1 and Figure 1). Twenty-nine regions were detected only in SSB and two only in the empty cup, showing a marked change in the aroma-active profile after emptying. The FD profile also differed considerably between the two samples. In SSB, the odourants with the highest FD factors (FD 2048) were mainly low- to medium-boiling fruity esters, including ethyl butanoate, ethyl 3-methylbutanoate, ethyl pentanoate and ethyl hexanoate. These compounds have previously been reported as key odourants in several baijiu aroma types [23,24,25]. In contrast, the FD factor of sotolon was 16 in SSB but reached 2048 in the empty cup, the highest FD level tested. Hydrocinnamic acid, benzeneacetic acid and octanoic acid each had an FD factor of 256 in the empty cup. 1-octen-3-one and trimethylpyrazine reached FD 128, while phenylethyl alcohol, ethyl 2-methylbutanoate, 2,3-dimethyl-5-ethylpyrazine and ethyl hexadecanoate reached FD 64.

The distribution of FD factors among compound classes further illustrated this change. Fruity esters accounted for most of the odourants with FD 2048 in SSB but were absent from the highest FD levels in the empty cup, consistent with their greater loss after emptying. In contrast, several organic acids, including octanoic, hydrocinnamic and benzeneacetic acids, as well as sotolon and several pyrazines, showed relatively high FD factors in the empty cup despite lower values in SSB. Pyrazines contribute nutty and roasted notes to SSB even at low concentrations [26]. Previous studies have also shown synergistic interactions among some pyrazines, which can lower their effective detection thresholds and enhance their sensory contribution [27,28]. Their relatively high boiling points may also favour prolonged persistence in the empty cup. This may partly explain the prolonged detection of some nutty and roasty odourants during the empty cup period (Table S1). Lactones and long-chain esters generally showed intermediate FD factors in both samples.

### 3.2. Temporal Dynamics of Aroma-Active Compounds During Empty Cup Aroma Persistence

**Figure 2:**
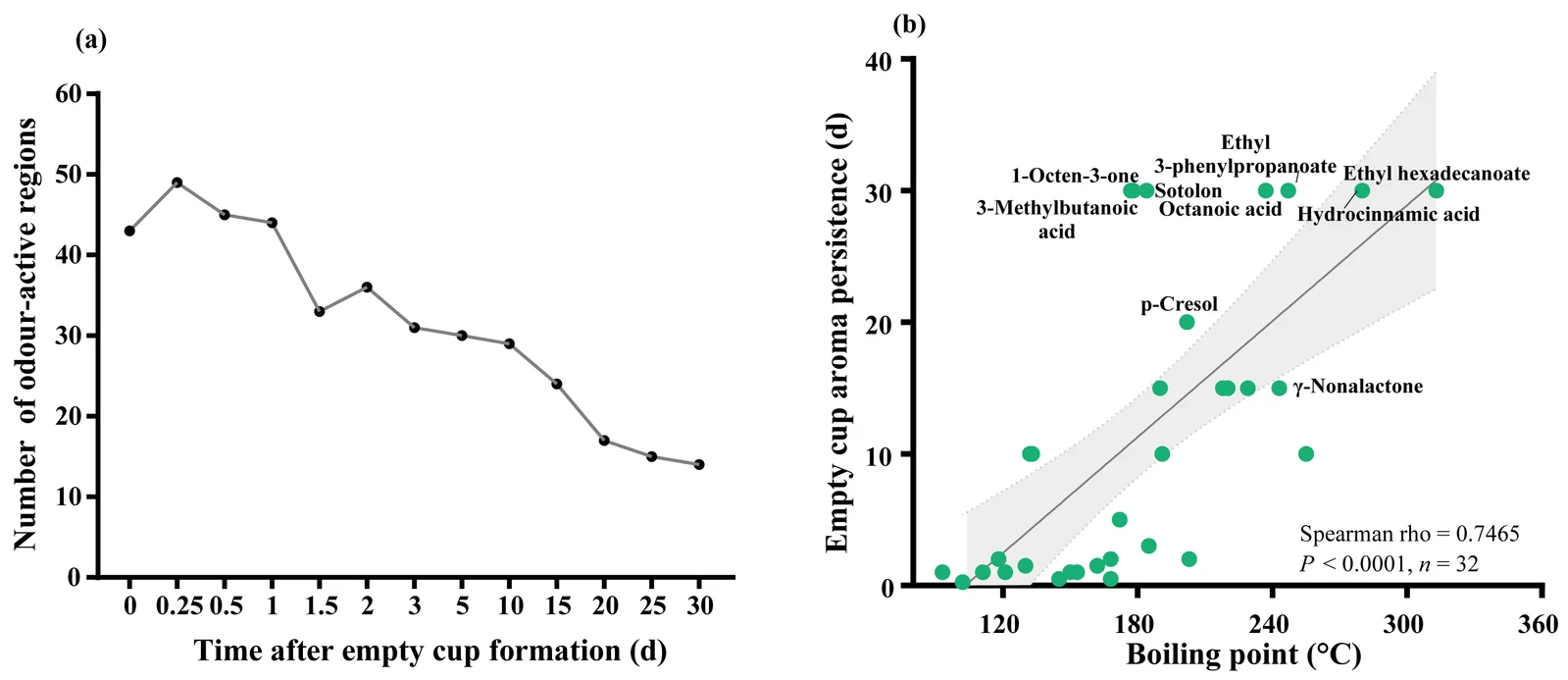
Temporal persistence of aroma-active compounds and its relationship with boiling point. (**a**) Number of aroma-active regions detected over 30 d after emptying. (**b**) Relationship between boiling point and aroma persistence for compounds with available boiling point data. Each dot represents one compound (*n* = 32), with selected compounds labeled directly on the plot.

The number of detectable aroma-active regions increased during the first 6 h and then generally declined over the 30 d period (Table S1 and Figure 2a). The number increased from 43 at 0 h to 49 at 6 h, then decreased to 45 at 12 h, 44 at 1 d and 33 at 1.5 d. A short plateau or partial rebound was observed between 2 and 5 d. Thereafter, the number declined from 29 at 10 d to 24 at 15 d, 17 at 20 d, 15 at 25 d and 14 at 30 d. The initial increase shows that the number of detectable odourants did not decrease monotonically immediately after emptying. One possible explanation is the relatively high ethanol concentration remaining immediately after emptying. Ethanol concentration can alter molecular interactions in ethanol–water systems and influence aroma partitioning, release and perception [29,30,31,32,33].

A study using a whisky model system showed that higher ethanol concentrations reduced the headspace partitioning of many aroma compounds [32]. In baijiu, ethanol concentration has also been shown to affect the olfactory thresholds and perception of individual odourants [33]. As ethanol evaporated from the residue, some odourants may therefore have become more readily extracted, which could partly explain the increase in detectable regions during the first 6 h. Because ethanol concentration was not measured during the experiment, this explanation remains tentative. After 6 h, the overall decline in detectable regions was consistent with the gradual loss of volatile compounds.

The 13-time-point, 30 d experiment extends previous studies that evaluated empty cup aroma at only one or a few time points [8,9,10,11]. Across the full time course, 49 aroma-active regions were detected (Table S1), of which 32 had available boiling point data for correlation analysis. This number was higher than the 36 regions detected by AEDA in the freshly emptied cup. The difference likely reflects the different sampling strategies. AEDA in Section 3.1 characterises the freshly emptied cup at a single time point using progressively increased thermal-desorption split ratios, whereas the time-course experiment examines residues at 13 time points over 30 d. The latter therefore captures regions detected at any stage of the persistence period, including some that were not readily detectable immediately after emptying but became detectable by GC-O at later time points as the composition of the residue changed. Fourteen regions remained detectable at 30 d. Identified compounds included 1-octen-3-one, sotolon, 3-methylbutanoic acid, ethyl 3-phenylpropanoate, octanoic acid, ethyl hexadecanoate and hydrocinnamic acid. Other odourants, including p-cresol, 2-acetylpyrrole, ethyl phenylacetate, phenylethyl alcohol, tetramethylpyrazine and γ-nonanolactone, remained detectable for 15–25 d. Long persistence was observed mainly among acids, nitrogen heterocycles, phenolic and other aromatic compounds, lactones and long-chain esters.

Several unidentified regions with roasty, nutty or seasoning-like descriptors also persisted for at least 15 d (Table S1). These sensory characteristics may be associated with pyrazines or other Maillard-derived odourants, but aroma descriptors alone are insufficient for compound identification. Further confirmation by complementary mass-spectrometric analysis would be required to establish their identities and contributions to persistent empty cup aroma.

### 3.3. Association Between Boiling Point and Empty Cup Aroma Persistence

Boiling point was strongly associated with persistence among the 32 compounds with available data (Spearman ρ = 0.7465, *p* < 0.0001; Table 2 and Figure 2b). Low-boiling compounds, including 3-methylbutyraldehyde (93 °C), acetal (102 °C), ethyl 2-methylpropanoate (111 °C) and ethyl butanoate (121 °C), remained detectable for only 0.25–1 d. By contrast, ethyl hexadecanoate (313 °C), hydrocinnamic acid (280 °C), ethyl 3-phenylpropanoate (247 °C), octanoic acid (237 °C), and sotolon (184 °C) remained detectable at 30 d.

Boiling point alone, however, did not fully account for persistence. Compounds with similar boiling points sometimes showed markedly different persistence. Ethyl 2-methylbutanoate and ethyl 3-methylbutanoate, with boiling points of 133 and 132 °C, respectively, remained detectable for 10 d, whereas 3-methyl-1-butanol (130 °C) persisted for only 1.5 d. Similarly, 1-octen-3-one (178 °C) remained detectable at the 30 d endpoint, whereas trimethylpyrazine (172 °C) persisted for only 5 d. An even clearer contrast was observed between sotolon (184 °C), which remained detectable at 30 d, and pentanoic acid (185 °C), which persisted for only 3 d. These examples show that compounds of comparable volatility can differ substantially in persistence. Previous studies have shown that matrix components such as lactic acid, ethyl lactate and other organic acids can influence the release of aroma compounds in Baijiu through molecular interactions [15,16,34]. More broadly, hydrogen-bond-driven self-assembly and other intermolecular associations have also been reported in Baijiu matrices [35,36]. Such interactions may partly explain why some compounds persisted longer than would be expected from boiling point alone.

Overall, these results suggest that empty cup aroma persistence is influenced by both compound volatility and interactions within the residual matrix. Boiling point provides a useful indicator of persistence, but the notable deviations observed for 1-octen-3-one and the two methylbutanoate esters indicate that volatility alone cannot fully account for their persistence. Because the association was evaluated using Spearman’s rank correlation and several persistence values were right-censored at the 30 d endpoint, a conventional regression-based residual analysis was not considered appropriate for the present dataset. Instead, the deviations were examined directly by comparing compounds with similar boiling points but different persistence times.

**Table 2:** Boiling points and persistence of 32 aroma-active compounds during the 30 d empty cup aroma experiment.

No.	Aroma Compound	Odour Descriptor	RI (DB-WAX)	Basis of Identification	Boiling Point (°C)	Aroma Persistence (d)	BP Source
1	Acetal	fruity	899	MS, aroma, RI, S	102	0.25	PubChem
2	3-Methylbutyraldehyde	green	917	MS, aroma, RI, S	93	1	PubChem
3	Ethyl 2-methylpropanoate	fruity	972	MS, aroma, RI, S	111	1	NIST
4	Ethyl butanoate	fruity	1046	MS, aroma, RI, S	121	1	NIST
5	Ethyl 2-methylbutanoate	berry	1062	MS, aroma, RI, S	133	10	NIST
6	Ethyl 3-methylbutanoate	fruity	1077	MS, aroma, RI, S	132	10	NIST
7	Ethyl pentanoate	fruity	1138	MS, aroma, RI, S	145	0.5	PubChem
8	3-Methyl-1-butanol	alcoholic	1220	MS, aroma, RI, S	130	1.5	NIST
9	Ethyl hexanoate	fruity	1241	MS, aroma, RI, S	168	2	NIST
10	Furfuryl ethyl ether	burnt	1288	MS, aroma, RI	150	1	PubChem
11	1-Octen-3-one	mushroom	1303	aroma, RI, S	178	≥30	ACS/CAS
12	Dimethyl trisulfide	cabbage	1383	MS, aroma, RI, S	168	0.5	PubChem
13	Trimethylpyrazine	roasty	1414	MS, aroma, RI, S	172	5	NIST
14	Acetic acid	vinegar	1449	MS, aroma, RI, S	118	2	PubChem
15	2,3-Dimethyl-5-ethylpyrazine	nutty	1472	MS, aroma, RI, S	191	10	PubChem
16	Tetramethylpyrazine	roasty	1485	MS, aroma, RI, S	190	15	NIST
17	2-Methylpropionic acid	cheesy	1567	MS, aroma, RI, S	153	1	PubChem
18	Butanoic acid	cheesy	1626	MS, aroma, RI, S	162	1.5	NIST
19	3-Methylbutanoic acid	rancid	1669	MS, aroma, RI, S	177	≥30	PubChem
20	Pentanoic acid	rancid	1736	aroma, RI, S	185	3	PubChem
21	Ethyl phenylacetate	floral	1789	MS, aroma, RI, S	229	15	NIST
22	Hexanoic acid	rancid	1844	MS, aroma, RI, S	203	2	PubChem
23	Ethyl 3-phenylpropanoate	floral	1889	MS, aroma, RI, S	247	≥30	NIST
24	Phenylethyl alcohol	rosy	1914	MS, aroma, RI, S	218	15	NIST
25	2-Acetylpyrrole	roasty	1978	aroma, RI, S	220	15	NIST
26	γ-Nonanolactone	coconut	2038	MS, aroma, RI, S	243	15	TCI
27	Octanoic acid	rancid	2042	aroma, RI, S	237	≥30	NIST
28	p-Cresol	animal	2078	aroma, RI, S	202	20	PubChem
29	Nonanoic acid	cheesy	2175	aroma, RI, S	255	10	PubChem
30	Sotolon	seasoning-like, herbal	2210	aroma, RI, S	184	≥30	Ref. [37]
31	Ethyl hexadecanoate	waxy	2261	MS, aroma, RI, S	313	≥30	Ref. [38]
32	Hydrocinnamic acid	cheesy	2624	aroma, RI, S	280	≥30	NIST

RI, retention index; MS, mass spectrum; aroma, aroma quality; S, authentic standard. Values reported as ≥30 d indicate compounds that remained detectable at the final observation and were therefore right-censored by the 30 d experimental window. Boiling point sources are listed for each compound. Literature-reported values were used for sotolon [37] and ethyl hexadecanoate [38]. Boiling point values refer to atmospheric-pressure values where available.

### 3.4. Two Temporal Patterns of Residue Peak Area Changes During Empty Cup Aroma Persistence

**Figure 3:**
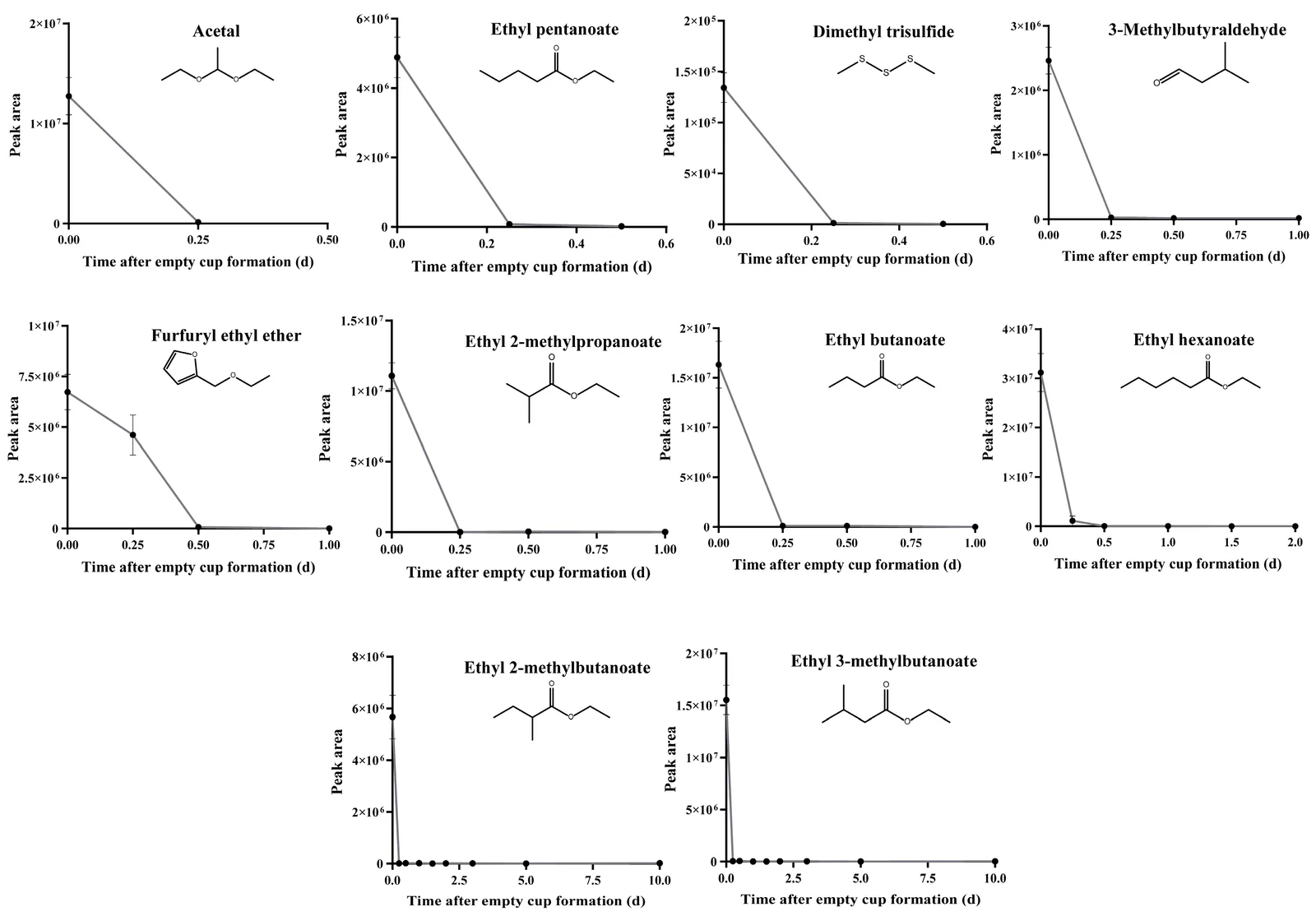
Representative aroma-active compounds showing rapid decreases in residue peak area during empty cup aroma persistence. Two-dimensional structures are shown for the indicated compounds.

**Figure 4:**
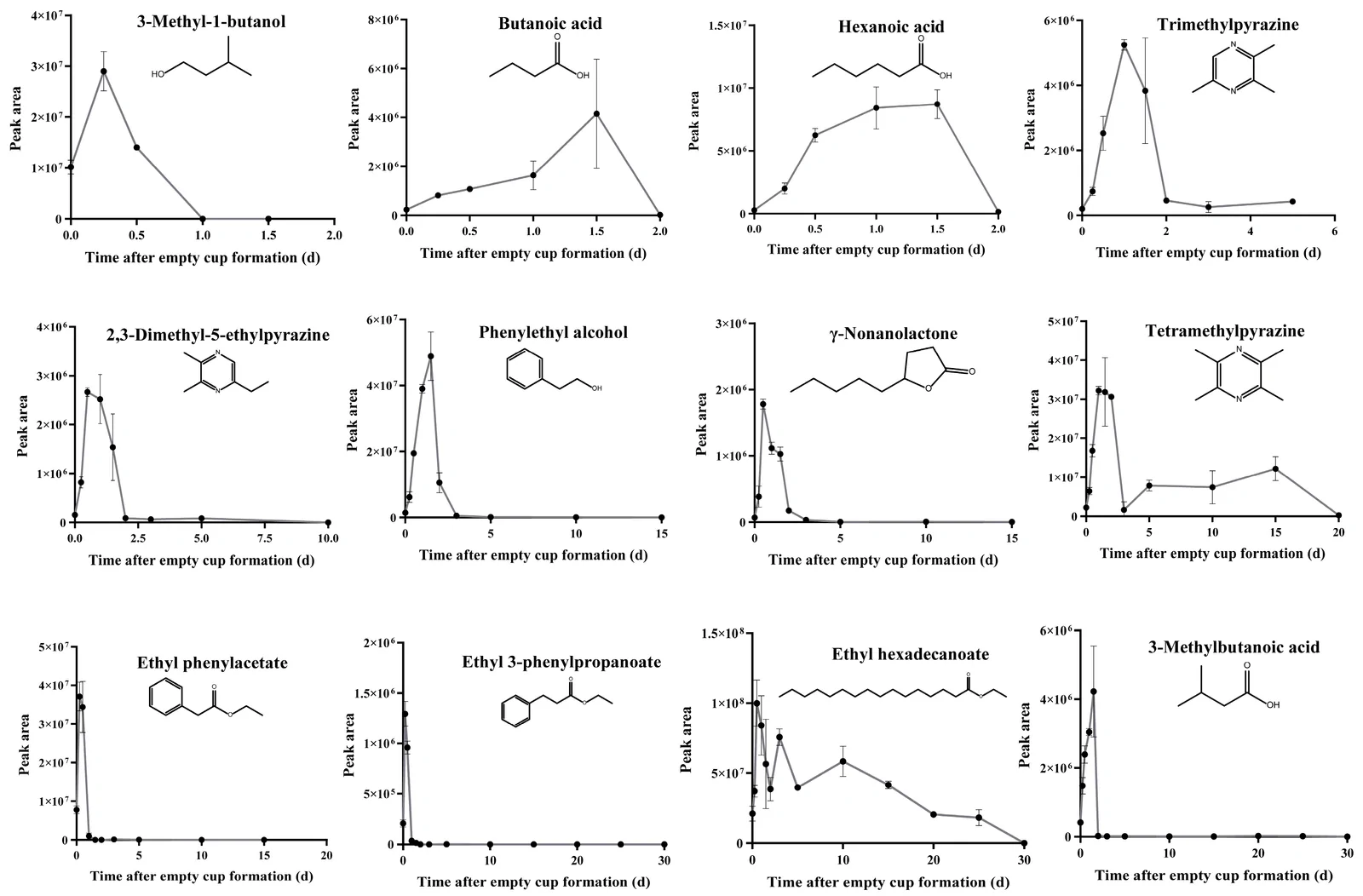
Representative aroma-active compounds showing delayed maxima or non-monotonic changes in residue peak area during empty cup aroma persistence. Two-dimensional structures are shown for the indicated compounds.

Figure 3 and Figure 4 show the residue peak area profiles of 22 identified aroma-active compounds whose chromatographic signals could be reliably resolved and tracked over the relevant time points. Aroma-active regions for which reliable peak-area tracking was not possible were not included in this analysis. Based on their temporal peak-area profiles, the 22 compounds were broadly grouped into two patterns. Ten compounds had their highest peak areas immediately after emptying and then declined. Acetal, dimethyl trisulfide and 3-methylbutyraldehyde decreased to near-zero levels by 6 h. Ethyl pentanoate, ethyl 2-methylpropanoate, ethyl butanoate, ethyl 2-methylbutanoate and ethyl 3-methylbutanoate also decreased rapidly. Ethyl hexanoate decreased to near-zero levels by 12 h, while furfuryl ethyl ether showed a similarly rapid decline. These chromatographic changes did not always correspond to the olfactory persistence endpoint reported in Table S1.

The other 12 compounds generally showed delayed maxima or non-monotonic changes after emptying. 3-Methyl-1-butanol, phenylethyl alcohol, γ-nonanolactone, ethyl phenylacetate, ethyl 3-phenylpropanoate and 3-methylbutanoic acid reached pronounced early maxima and then decreased rapidly. Butanoic acid and hexanoic acid increased more gradually and reached their highest peak areas at approximately 1.5 d. Trimethylpyrazine and 2,3-dimethyl-5-ethylpyrazine also peaked early but remained detectable at low levels for several days. Tetramethylpyrazine and ethyl hexadecanoate showed more complex, non-monotonic profiles. Overall, both the timing of the maximum peak area and the subsequent decline differed among compounds, and changes in chromatographic peak area did not always correspond to sensory persistence.

The rapid decline of some compounds may reflect their limited retention in the residual matrix after emptying. In contrast, delayed maxima may result from changes in residue composition as ethanol and water evaporate, which could alter interactions between odourants and less volatile matrix components [39]. This interpretation remains tentative because SBSE measures compounds recovered from the residue rather than their concentrations in the headspace [40]. The peak area profiles therefore reflect temporal changes in the residual material but cannot directly demonstrate delayed headspace release or first-order evaporation behaviour.

### 3.5. Integration of Odour Potency and Persistence Identifies Candidate Empty Cup Markers

Candidate contributors were evaluated by considering FD factor, persistence and temporal peak area patterns together. Sotolon, octanoic acid, hydrocinnamic acid, 1-octen-3-one and ethyl hexadecanoate combined relatively high FD factors in the empty cup with prolonged persistence. Ethyl 3-phenylpropanoate and 3-methylbutanoic acid were also notable because they remained detectable at 30 d and showed delayed or non-monotonic peak area changes. These compounds may therefore serve as candidate markers of persistent empty cup aroma.

In contrast, several odourants with high FD factors in SSB showed weak or short-lived signals in the empty cup. Ethyl butanoate, ethyl pentanoate, ethyl hexanoate and dimethyl trisulfide were typical examples. Their rapid decline illustrates the marked difference between the aroma-active profiles of fresh SSB and the empty cup. Other compounds, including p-cresol, phenylethyl alcohol and γ-nonanolactone, had moderate FD factors but persisted for relatively long periods. Their actual contributions to empty cup aroma require further validation using complementary quantitative and sensory approaches, including odour activity values, aroma recombination, omission experiments and temporal sensory evaluation [41,42,43,44].

Taken together, these results show that no single measure is sufficient to evaluate persistent empty cup odourants. Considering odour potency, persistence and temporal peak area changes together help distinguish compounds that are prominent shortly after emptying from those that remain detectable for longer periods. The 30 d time-course experiment therefore provides additional information beyond measurements made at a single time point and shows when different odourants are most prominent during empty cup aroma persistence.

This study has two main limitations. First, the investigation was conducted using one commercial SSB sample. Although the replicated time-course design allowed detailed characterisation of temporal changes within this sample, FD rankings and persistence patterns may differ among SSBs from different producers, batches and production conditions. Future studies including a broader range of SSB samples would help evaluate the general applicability of these findings. Second, glass headspace vials were used to simulate empty cups under controlled experimental conditions. Because cup material may influence odourant adsorption and retention, comparison with porcelain, ceramic and other commonly used tasting vessels would be useful to further assess the effect of vessel material on aroma persistence.

## 4. Conclusions

This study shows that the persistent empty cup aroma of soy sauce aroma type baijiu (SSB) is associated with selective retention and marked changes in the aroma-active profile after emptying. AEDA detected 65 aroma-active regions across SSB and the empty cup, including 63 in SSB, 36 in the empty cup and 34 shared by both. The FD profile changed substantially after emptying, with fruity esters becoming less prominent and sotolon, several acids and pyrazines showing greater sensory potency in the empty cup. The FD factor of sotolon was 16 in SSB and reached the highest tested level of 2048 in the empty cup, whereas several high FD esters in SSB declined rapidly after emptying.

Time-resolved monitoring showed that the number of detectable aroma-active regions initially increased from 43 at 0 h to 49 at 6 h and then gradually decreased, with 14 regions still detectable at 30 d. Persistence was strongly associated with boiling point (ρ = 0.7465, *p* < 0.0001, *n* = 32), although several compounds deviated from this general relationship, indicating that volatility alone cannot fully explain odourant persistence. Considering FD factor, persistence and temporal peak area patterns together identified sotolon, several organic acids, 1-octen-3-one and selected esters as candidate markers of persistent empty cup aroma. These findings show that empty cup aroma changes over time after the baijiu is removed and that different odourants contribute at different stages of the persistence period. Further studies using additional SSB samples, different cup materials and quantitative sensory validation would help further evaluate the general applicability and sensory relevance of these findings.

## Figures and Tables

**Table 1:** Aroma-active regions detected by SBSE-GC-O-MS and their FD factors determined by AEDA in soy sauce aroma type baijiu (SSB) and the empty cup.

No.	Aroma Compound	Odour Descriptor	RI	Basis of Identification	FD Factor	
			DB-WAX		SSB	Empty Cup
1	2-Methylpropanal	malt	809	MS, aroma, RI, S	8	—
2	Ethyl acetate	fruity	895	MS, aroma, RI, S	256	—
3	3-Methylbutyraldehyde	green	926	MS, aroma, RI, S	1024	—
4	Ethyl 2-methylpropanoate	fruity	988	MS, aroma, RI, S	1024	4
5	Ethyl butanoate	fruity	1053	MS, aroma, RI, S	2048	4
6	Ethyl 2-methylbutanoate	berry	1068	MS, aroma, RI, S	1024	64
7	Ethyl 3-methylbutanoate	fruity	1085	MS, aroma, RI, S	2048	2
8	2-Methyl-1-propanol	grass	1096	MS, aroma, RI, S	4	—
9	3-Methylbutyl acetate	banana	1135	MS, aroma, RI, S	4	—
10	Ethyl pentanoate	fruity	1154	MS, aroma, RI, S	2048	—
11	unknown	alcoholic	1190	aroma	64	—
12	Ethyl 4-methylpentanoate	fruity	1200	MS, aroma, RI, S	64	—
13	3-Methyl-1-butanol	alcoholic	1238	MS, aroma, RI, S	512	—
14	Ethyl hexanoate	fruity	1258	MS, aroma, RI, S	2048	—
15	Furfuryl ethyl ether	burnt	1296	MS, aroma, RI	2048	—
16	1-Octen-3-one	mushroom	1312	aroma, RI, S	16	128
17	2,6-Dimethylpyrazine	nutty	1323	MS, aroma, RI, S	32	2
18	Ethyl lactate	fruity	1336	MS, aroma, RI, S	8	—
19	unknown	roasty	1370	aroma	16	—
20	Dimethyl trisulfide	cabbage	1393	MS, aroma, RI, S	1024	—
21	2-Ethyl-6-methylpyrazine	nutty	1400	MS, aroma, RI, S	—	2
22	unknown	cheesy	1413	aroma	128	—
23	Trimethylpyrazine	roasty	1423	MS, aroma, RI, S	4	128
24	2-Furanmethanethiol	roasty	1434	aroma, RI, S	256	—
25	unknown	nutty	1449	aroma	1024	4
26	Acetic acid	vinegar	1460	MS, aroma, RI, S	128	8
27	2,3-Dimethyl-5-ethylpyrazine	nutty	1481	MS, aroma, RI, S	64	64
28	Tetramethylpyrazine	roasty	1494	MS, aroma, RI, S	32	16
29	2,3-Butanediol	vegetable	1543	MS, aroma, RI, S	4	2
30	unknown	cucumber	1598	aroma	64	—
31	2-Undecanone	fatty	1614	MS, aroma, RI	4	—
32	Butanoic acid	cheesy	1638	MS, aroma, RI, S	8	16
33	2-Acetyl-5-methylfuran	roasty	1644	aroma, RI, S	16	2
34	1-Nonanol	green	1656	MS, aroma, RI, S	8	—
35	3-Methylbutanoic acid	rancid	1679	aroma, RI, S	256	32
36	unknown	roasty	1684	aroma	1024	—
37	unknown	roasty	1697	aroma	64	—
38	Benzene,(2,2-diethoxyethyl)-	vegetable	1712	MS, aroma, RI	8	—
39	Pentanoic acid	rancid	1749	MS, aroma, RI, S	4	8
40	unknown	coriander	1771	aroma	16	—
41	Ethyl phenylacetate	floral	1808	MS, aroma, RI, S	128	—
42	unknown	mouldy	1827	aroma	4	8
43	β-Damascenone	honey	1847	aroma, RI, S	1024	2
44	Hexanoic acid	rancid	1856	MS, aroma, RI, S	16	2
45	Geranylacetone	floral	1876	MS, aroma, RI, S	4	4
46	Ethyl 3-phenylpropanoate	floral	1902	MS, aroma, RI, S	1024	16
47	Phenylethyl alcohol	rosy	1927	MS, aroma, RI, S	128	64
48	Heptanoic acid	cheesy	1962	MS, aroma, RI, S	2	2
49	2-Acetylpyrrole	roasty	1986	MS, aroma, RI, S	512	16
50	γ-Nonanolactone	coconut	2049	MS, aroma, RI, S	128	32
51	Octanoic acid	rancid	2053	MS, aroma, RI, S	512	256
52	unknown	floral	2089	aroma	256	—
53	p-Cresol	animal	2093	MS, aroma, RI, S	32	32
54	3-Phenylthiophene	roasty	2151	MS, aroma, RI	1024	—
55	unknown	milk	2167	aroma	4	—
56	Nonanoic acid	cheesy	2177	aroma, RI, S	4	—
57	unknown	burnt	2194	aroma	4	8
58	Sotolon	seasoning-like, herbal	2213	aroma, RI, S	16	2048
59	Ethyl hexadecanoate	waxy	2269	MS, aroma, RI, S	128	64
60	n-Decanoic acid	soapy	2284	MS, aroma, RI, S	4	—
61	unknown	roasty	2299	aroma	2	8
62	unknown	herbal	2372	aroma	—	64
63	unknown	fatty	2598	aroma	256	64
64	Benzeneacetic acid	floral	2600	MS, aroma, RI, S	32	256
65	Hydrocinnamic acid	cheesy	2665	MS, aroma, RI, S	128	256

RI, retention index; FD, flavour dilution factor; MS, mass spectrum; aroma, aroma quality; S, authentic standard; —, not detected.

## Data Availability

The original contributions presented in this study are included in the article/Supplementary Materials. Further inquiries can be directed to the corresponding author.

## Supplementary Materials

The following supporting information can be downloaded at https://www.mdpi.com/article/10.3390/foods15162906/s1, Table S1. Time-resolved aroma intensity profiles of 49 aroma-active regions during empty cup aroma persistence over 30 d.
