# Supplementary material for: Time-Resolved Profiling of Aroma-Active Compounds During the Persistence of Empty Cup Aroma in Soy Sauce Aroma Type Baijiu
Source: Foods. 2026 Aug 19;15(16):2906. doi: 10.3390/foods15162906 (PMC13512373; doi:10.3390/foods15162906)
Supplement: Supplementary file 1 [file foods-15-02906-s001.zip › foods-4473195-supplementary.pdf]

Supplementary Materials

Table S1. Time-resolved aroma intensity profiles of 49 aroma-active regions during empty cup aroma persistence over 30 d.

No.	Aroma compounds	Descriptor	RI (DB-WAX)	Basis of ID	0 d	0.25 d	0.5 d	1 d	1.5 d	2 d	3 d	5 d	10 d	15 d	20 d	25 d	30 d
1	Acetal	fruity	899	MS, aroma, RI, S	2.3	1.0	—	—	—	—	—	—	—	—	—	—	—
2	3-Methylbutyraldehyde	green	917	MS, aroma, RI, S	3.7	1.0	1.0	2.0	—	—	—	—	—	—	—	—	—
3	Ethyl 2-methylpropanoate	fruity	972	MS, aroma, RI, S	4.0	1.0	1.5	1.5	—	—	—	—	—	—	—	—	—
4	Ethyl butanoate	fruity	1046	MS, aroma, RI, S	4.0	3.0	1.0	1.0	—	—	—	—	—	—	—	—	—
5	Ethyl 2-methylbutanoate	berry	1062	MS, aroma, RI, S	4.0	2.0	2.5	1.5	2.0	1.0	1.0	1.0	2.0	—	—	—	—
6	Ethyl 3-methylbutanoate	fruity	1077	MS, aroma, RI, S	2.5	2.0	—	2.0	—	1.5	1.0	1.5	1.5	—	—	—	—
7	Ethyl pentanoate	fruity	1138	MS, aroma, RI, S	2.5	2.7	1.0	—	—	—	—	—	—	—	—	—	—
8	3-Methyl-1-butanol	alcoholic	1220	MS, aroma, RI, S	2.5	4.0	3.7	—	2.0	—	—	—	—	—	—	—	—
9	Ethyl hexanoate	fruity	1241	MS, aroma, RI, S	2.5	1.5	—	1.0	—	1.0	—	—	—	—	—	—	—
10	unknown	burnt	1259	aroma	2.0	1.0	1.0	1.0	—	—	—	—	—	—	—	—	—
11	Furfuryl ethyl ether	burnt	1288	MS, RI	3.5	3.0	2.0	3.0	—	—	—	—	—	—	—	—	—
12	1-Octen-3-one	mushroom	1303	aroma, RI, S	2.0	1.7	1.0	2.0	1.0	1.0	1.0	1.0	1.0	1.0	1.7	1.0	1.3
13	unknown	roasty	1318	aroma	2.5	2.3	3.0	3.3	3.3	3.0	2.0	3.0	—	—	—	—	—
14	unknown	roasty	1377	aroma	1.0	1.5	—	1.0	2.0	2.0	—	1.0	—	—	—	—	—
15	Dimethyl trisulfide	cabbage	1383	MS, aroma, RI, S	3.5	1.5	1.0	—	—	—	—	—	—	—	—	—	—
16	Trimethylpyrazine	nutty	1414	MS, aroma, RI, S	2.0	2.3	3.0	3.0	3.0	2.5	2.0	1.7	—	—	—	—	—
17	unknown	roasty	1435	aroma	3.0	2.7	2.3	3.0	3.0	3.0	2.0	2.0	1.3	1.0	1.0	1.0	1.0

No.	Aroma compounds	Descriptor	RI (DB-WAX)	Basis of ID	0 d	0.25 d	0.5 d	1 d	1.5 d	2 d	3 d	5 d	10 d	15 d	20 d	25 d	30 d
18	Acetic acid	vinegar	1449	MS, aroma, RI, S	—	1.3	1.7	1.5	—	2.0	—	—	—	—	—	—	—
19	2,3-Dimethyl-5-ethylpyrazine	nutty	1472	MS, aroma, RI, S	2.5	3.0	3.0	3.0	3.0	1.7	2.0	1.5	1.0	—	—	—	—
20	Tetramethylpyrazine	roasty	1485	MS, aroma, RI, S	1.7	2.0	1.0	2.0	1.5	1.0	—	1.5	1.0	1.5	—	—	—
21	unknown	moldy	1514	aroma	1.0	1.7	1.5	1.3	1.7	2.0	2.0	2.5	2.0	2.0	1.0	—	—
22	unknown	moldy	1541	aroma	3.0	2.3	3.0	2.7	2.5	2.0	2.0	2.0	2.0	1.0	1.0	1.3	1.5
23	2-Methylpropionic acid	cheesy	1567	MS, aroma, RI, S	1.0	1.3	1.7	1.7	—	—	—	—	—	—	—	—	—
24	unknown	cucumber	1589	aroma	1.5	1.7	2.0	2.0	1.5	2.0	—	—	—	—	—	—	—
25	Butanoic acid	cheesy	1626	MS, aroma, RI, S	2.0	2.3	2.3	2.7	2.7	—	—	—	—	—	—	—	—
26	unknown	roasty	1633	aroma	2.0	2.0	2.0	1.7	2.0	1.7	2.3	2.5	2.3	1.7	1.5	1.5	1.0
27	3-Methylbutanoic acid	rancid	1669	MS, aroma, RI, S	2.7	3.7	3.0	3.0	3.0	2.0	1.5	1.3	2.0	2.0	2.0	2.0	2.0
28	unknown	nutty	1678	aroma	3.5	2.7	1.3	2.5	2.0	1.5	—	1.5	1.0	—	—	—	—
29	Pentanoic acid	rancid	1736	aroma, RI, S	—	1.5	1.5	2.0	1.7	1.0	1.0	—	—	—	—	—	—
30	Ethyl phenylacetate	floral	1789	MS, aroma, RI, S	2.0	2.7	2.7	1.0	—	2.0	1.0	1.0	—	2.0	—	—	—
31	unknown	seasonings	1814	aroma	3.0	2.3	2.7	2.7	2.0	2.0	2.0	2.0	2.0	2.0	2.0	2.0	2.0
32	unknown	honey	1834	aroma	4.0	3.3	3.3	2.0	3.0	1.3	1.5	—	2.0	—	—	—	—
33	Hexanoic acid	rancid	1844	MS, aroma, RI, S	—	2.7	2.7	3.0	3.0	1.0	—	—	—	—	—	—	—
34	Ethyl 3-phenylpropanoate	floral	1889	MS, aroma, RI, S	3.5	3.7	3.0	2.3	1.0	2.3	1.7	2.0	1.7	1.0	1.0	1.0	1.5
35	Phenylethyl alcohol	rosy	1914	MS, aroma, RI, S	1.7	2.0	2.0	2.0	2.0	2.0	2.0	1.7	2.0	1.0	—	—	—
36	2-Acetylpyrrole	roasty	1978	aroma, RI, S	3.0	3.0	2.5	2.0	1.0	—	1.0	2.0	1.0	2.5	—	—	—
37	unknown	watery	2004	aroma	—	1.0	1.0	1.3	1.0	1.0	1.7	1.3	1.0	1.0	1.0	1.0	1.0
38	γ-Nonanolactone	coconut	2038	MS, aroma, RI, S	2.0	2.0	3.0	3.0	2.0	2.3	2.5	2.0	2.0	3.0	—	—	—

No.	Aroma compounds	Descriptor	RI (DB-WAX)	Basis of ID	0 d	0.25 d	0.5 d	1 d	1.5 d	2 d	3 d	5 d	10 d	15 d	20 d	25 d	30 d
39	Octanoic acid	rancid	2042	aroma, RI, S	2.0	2.7	2.0	2.0	2.3	2.7	2.3	1.3	1.0	2.0	1.0	1.0	1.5
40	p-Cresol	animal	2078	aroma, RI, S	1.0	2.0	2.7	2.0	1.7	2.0	1.5	2.0	1.0	2.5	1.0	—	—
41	unknown	flour	2136	aroma	1.5	1.7	1.7	1.5	—	—	2.0	2.0	1.0	2.0	1.0	1.0	—
42	unknown	roasty	2164	aroma	2.0	3.0	2.0	—	—	2.0	2.0	2.0	1.0	2.0	2.0	1.3	2.0
43	Nonanoic acid	cheesy	2175	aroma, RI, S	1.5	1.0	1.3	1.3	1.0	1.0	1.0	—	1.0	—	—	—	—
44	Sotolon	seasoning-like, herbal	2210	aroma, RI, S	1.3	2.0	3.3	3.7	4.0	4.0	3.7	4.0	4.0	3.3	3.7	4.0	4.0
45	Ethyl hexadecanoate	waxy	2261	MS, aroma, RI, S	—	2.7	2.7	2.3	3.0	2.3	1.5	2.0	1.0	1.3	1.0	1.0	1.0
46	unknown	herbal	2363	aroma	—	1.0	2.0	1.3	—	1.0	1.0	2.0	2.0	1.0	—	—	—
47	unknown	fatty	2598	aroma	3.0	3.3	3.3	3.3	2.7	3.3	4.0	3.3	2.0	1.0	—	—	—
48	unknown	floral	2600	aroma	1.0	2.0	2.3	2.3	2.3	3.0	2.7	3.0	3.0	2.7	1.7	2.3	2.0
49	Hydrocinnamic acid	cheesy	2624	aroma, RI, S	1.0	2.5	2.7	2.7	3.0	3.0	3.3	3.3	4.0	3.0	3.0	3.0	2.3

Notes: Values are mean aroma intensity scores. —, not detected. Aroma persistence is the last sampling time meeting the predefined detection criterion. MS, mass spectrometry; RI, retention index; S, authentic standard.
